# Towards a general growth model for graphene CVD on transition metal catalysts†
†Electronic supplementary information (ESI) available: Fig. S1. See DOI: 10.1039/c5nr06873h



**DOI:** 10.1039/c5nr06873h

**Published:** 2016-01-05

**Authors:** Andrea Cabrero-Vilatela, Robert S. Weatherup, Philipp Braeuninger-Weimer, Sabina Caneva, Stephan Hofmann

**Affiliations:** a Department of Engineering , University of Cambridge , Cambridge CB3 0FA , UK . Email: rsw31@cam.ac.uk

## Abstract

The chemical vapour deposition (CVD) of graphene on three polycrystalline transition metal catalysts, Co, Ni and Cu, is systematically compared and a first-order growth model is proposed which can serve as a reference to optimize graphene growth on any elemental or alloy catalyst system. Simple thermodynamic considerations of carbon solubility are insufficient to capture even basic growth behaviour on these most commonly used catalyst materials, and it is shown that kinetic aspects such as carbon permeation have to be taken into account. Key CVD process parameters are discussed in this context and the results are anticipated to be highly useful for the design of future strategies for integrated graphene manufacture.

## Abstract 2

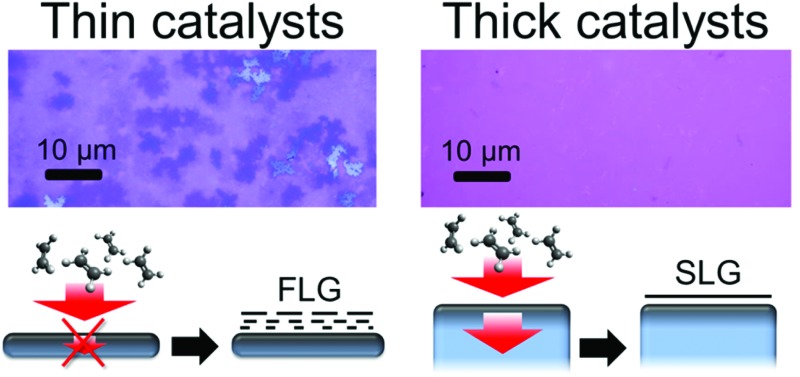
A first-order model for graphene CVD on transition metal catalysts that combines kinetic and thermodynamic considerations is developed and experimentally verified.

## Introduction

Chemical vapour deposition (CVD) has emerged as the dominant method to synthesise large single-crystalline domains and continuous films of “electronic-grade” graphene and other two-dimensional (2D) materials.^1–5^ Critical to the graphene CVD process is the use of a catalyst that enables low activation energy pathways for precursor dissociation, graphene nucleation, domain growth and merging. The question of which catalysts can be used is not only of fundamental importance but also a key issue for integrated graphene manufacture. Roll-to-roll approaches demand reusability of the catalyst, low cost and ease of graphene removal,^6–8^ whereas approaches without graphene transfer where the catalyst is part of the device structure or removed while the graphene stays on the target substrate demand graphene growth on a wide range of different catalyst materials at suitably benign CVD conditions.^9,10^ In spite of numerous surface science studies on the atomic structure of graphene layers on different single-crystal metal surfaces,^11,12^ the conditions and challenges for scalable graphene CVD are notably distinct and with a lack of basic understanding of graphene formation, catalyst selection criteria are yet to be well established.

Here, we systematically compare graphene CVD on three polycrystalline transition metal catalysts, namely Co, Ni and Cu, and propose a first-order growth model that can serve as a reference to optimize growth on any elemental or alloy catalyst system. A rationale widely adopted in current literature is that a catalyst material with low carbon solubility, such as Cu, is necessary for single layer growth^13,14^ to avoid graphene layer formation by carbon precipitation on cooling.^15–18^ Uniform growth of single layer graphene (SLG) on polycrystalline catalysts with significantly higher carbon solubility, including Ni^19^ and Pt,^20^ can, however, be routinely demonstrated,^21,22^ and it has been shown that single and few-layer graphene growth in particular for lower process temperatures occurs predominantly during hydrocarbon exposure at temperature and not during cooling.^11,23,24^ While simple thermodynamic considerations of carbon solubility are insufficient to adequately describe even basic growth behaviour on these most commonly used catalyst materials, we show that kinetic aspects such as carbon permeation have to be taken into account. Our simple model allows us to highlight key CVD parameters including the catalyst thickness alongside the growth temperature, carbon precursor pressure, exposure time and cooling rate. This captures well the behaviour apparent in our graphene CVD calibrations on Co, Ni and Cu and we expect our results to be highly useful for the design of future strategies for integrated graphene manufacture.

## Results

**Fig. 1:**
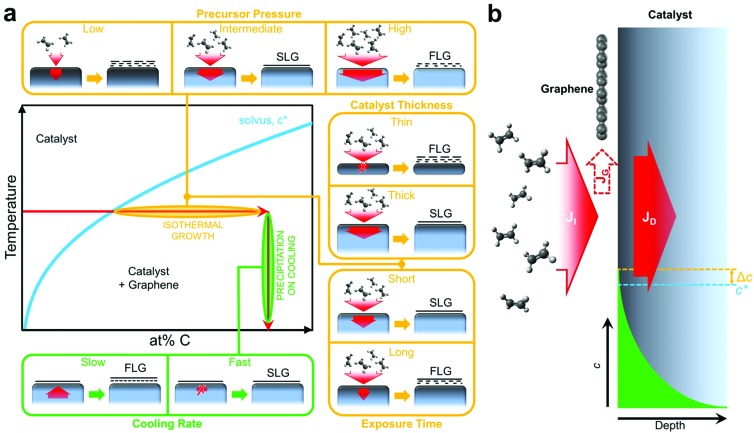
(a) Simple C-metal solid solution phase diagram of the catalyst surface showing two possible routes for graphene growth: isothermal and precipitation. Yellow boxes illustrate the effects of kinetic parameters (precursor partial pressure – top inset, catalyst thickness – right-top inset, exposure time – right-bottom inset) during isothermal growth. Bottom green inset illustrates the role of cooling rate for growth by precipitation on cooling. (b) Schematic showing the relation between the C flux resulting from precursor impingement and its dissociation at the catalyst surface (*J*_I_), the C diffusion into the catalyst (*J*_D_) and the graphene formation (*J*_G_) where the latter results from the difference between *J*_I_ and *J*_D_.

The general growth scenario of catalytic graphene CVD is schematically outlined in Fig. 1. Graphene nucleation and subsequent growth requires a carbon supersaturation at the catalyst surface. As indicated in the generic phase diagram section of Fig. 1a, such supersaturation can result from the solvus being crossed horizontally *via* continued hydrocarbon exposure and dissociation at the catalyst surface at constant temperature, which we refer to as isothermal growth, or it can also be crossed vertically at a given carbon concentration *via* catalyst cooling and the reduction in carbon solubility, which we refer to as precipitation on cooling. For a basic CVD process, consisting of heating up and pre-treatment of a catalyst (annealing in reducing gas), exposure to a hydrocarbon at constant temperature and cooling down in an inert atmosphere, graphene formation typically proceeds *via* isothermal growth but additional growth may also occur during cooling. Hence it is often argued that for the CVD of SLG, a catalyst with low carbon solubility is essential, and that for high carbon solubility metals, additional layers grow by precipitation upon cooling leading to multilayer formation.^13–18^ Fig. 1b shows why such simple thermodynamic considerations of carbon solubility are insufficient to capture even basic growth behaviour. The central point is that while the catalyst's carbon solubility presents a potential reservoir, depending on CVD conditions, this reservoir may never be filled, and thus the kinetics of the CVD process are critical to the growth behaviour. A basic balance can thus be considered between the carbon flux due to precursor impingement and dissociation, *J*_I_, and that related to carbon diffusion into the catalyst, *J*_D_, with the difference in fluxes, *J*_G_, feeding the growing graphene layer (Fig. 1b).

The insets of Fig. 1a outline the effect of key CVD parameters in terms of this kinetic carbon flux balance. Intermediate precursor (partial) pressures can result in a local carbon supersaturation developing at the catalyst surface that leads to SLG formation, whilst the extent of carbon diffusion into the catalyst bulk remains limited. Hence complete, SLG coverage can be achieved on a thick, high carbon solubility catalyst without it becoming saturated throughout with carbon.^19^ Too high precursor (partial) pressures lead to the direct nucleation of multilayer graphene due to a high supersaturation developing at the catalyst surface. Low precursor (partial) pressures relative to the permeability (the product of solubility, *S*, and diffusivity, *D*) of carbon in the catalyst will lead to an effective filling of the catalyst bulk with carbon, which in turn can precipitate depending on cooling rate (see below). The thickness of the catalyst dictates the size of the potential carbon reservoir and hence plays an important role in this kinetic model. A thick catalyst will allow continued carbon diffusion into the bulk, *J*_D_, which acts as mediating sink for carbon *via* the flux balance between *J*_I_, *J*_D_ and *J*_G_ at the catalyst surface and hence provides robust conditions for isothermal SLG growth. The bulk of thinner catalysts will quickly saturate throughout with carbon, rapidly lowering *J*_D_ and leading to FLG formation and inhomogeneous growth as *J*_I_ ≫ *J*_D_. Exposure time to the carbon precursor is another important parameter. For SLG CVD conditions, too short exposures lead to isolated graphene domains, whereas too long exposure leads to the isothermal formation of additional layers even after complete SLG coverage has been achieved. In this context, catalytic graphene CVD is often described as inherently being self-limited, since coverage of the catalyst with SLG will lower *J*_I_. However, this only refers to conditions of low chemical potential *i.e.* low precursor pressures where a window of exposure time exists over which SLG can be uniformly stabilised. For typical catalyst metals it can be shown (see below), that at higher precursor pressures and/or longer growth times additional graphene layers nucleate at the interface between the catalyst and the initial graphene layer fed through intrinsic defects (including grain boundaries) in the initial SLG. This presents a possible pathway for the controlled CVD of bi- and tri-layer graphene films. The effect of catalyst cooling will depend on the amount of carbon in the catalyst bulk. For slow cooling rates, the direction of *J*_D_ will reverse and assuming sufficient carbon in the catalyst bulk additional graphene layers can form. For fast cooling rates, however, *J*_D_ will be suppressed and the carbon will remain in the catalyst bulk, *i.e.* a high cooling rate helps prevent the precipitation of additional layers upon cooling.

The growth model outlined in Fig. 1 can be effectively applied to any elemental or alloy catalyst system, as we highlight in the following by comparing graphene CVD from high carbon solubility catalysts, Co (∼0.13 atom% at 700 °C)^25^ and Ni (∼0.19 atom% at 600 °C),^26^ to a common low carbon solubility catalyst, Cu (0.0007–0.0280 atom% at 1000 °C).^27,28^ For the latter it should be noted that while the carbon solubility is relatively low (reported values widely vary), there is little data available on the diffusivity of carbon in Cu,^27^ meaning that significant carbon permeation into thicker Cu may be possible, and thus the role of the catalyst bulk still needs to be considered. This is also relevant to growth strategies that target graphene growth at the back interface of the catalyst, *i.e.* growth at the catalyst-substrate interface or growth based on catalyst foil pockets.^29–32^

**Fig. 2:**
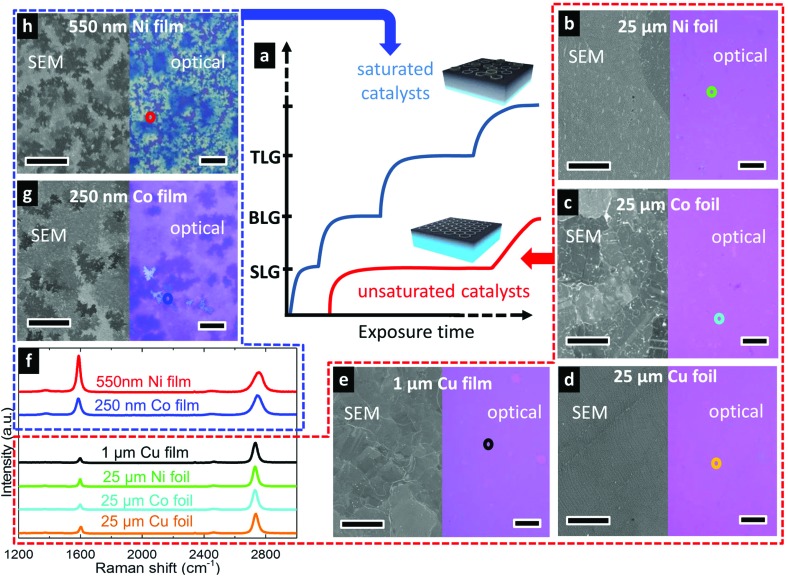
(a) Plot showing graphene growth evolution in terms of number of layers formed for catalysts saturated throughout their thickness with carbon (saturated catalysts) and catalysts that are only filled with carbon close to their surface and not throughout their thickness (unsaturated catalysts). (b–e, g, h) SEM images before transfer, and optical micrographs following transfer to SiO_2_(300 nm)/Si substrates, of graphene grown on Ni 25 μm foil (b) and 550 nm film (h) [600 °C, 3 × 10^–5^ mbar, 15 min, C_2_H_2_]; Co 25 μm foil (c) and 250 nm film (g) [700 °C, 10^–6^ mbar 15 min then ∼10^–5^ mbar 5 min, C_2_H_2_]; Cu 25 μm (d) and 1 μm film (e) [1035 °C, 250 mbar, 120 min, CH_4_(0.012 sccm)/Ar(250 sccm)/H_2_(26 sccm)]. All scale bars are 10 μm. (f) Raman spectra (457 nm excitation) taken for each of the transferred graphene layers with the colours of spectra corresponding to the locations marked with circles in b–e, g, h at which the spectra were measured.

We investigate here the growth of graphene on commercially available polycrystalline foils and SiO_2_-supported sputter-deposited films of Co, Cu, and Ni as catalysts, and adopt a relatively simple CVD process^24^ in which these are heated to growth temperature in a H_2_-containing atmosphere, exposed to a hydrocarbon precursor (C_2_H_2_, CH_4_), and then cooled to room temperature directly following removal of the precursor (see Methods for catalyst specific details). Fig. 2 compares graphene layer formation grouped into carbon saturated and unsaturated catalysts. The blue line in Fig. 2a illustrates the growth evolution of catalysts that are saturated with carbon throughout their thickness where the nucleation of each additional layer occurs shortly after the completion of the previous layer (represented as short steps in the plot). The incubation time for the nucleation of each additional layer is relatively short as there is no mediating diffusion into the catalyst bulk (*J*_D_ ≈ 0, *J*_G_ ≈ *J*_I_) and thus the supersaturation necessary to nucleate the new layer is rapidly reached. In contrast, the red line shows graphene growth evolution for unsaturated catalysts. In this case, the catalyst bulk provides a sink into which carbon arriving at the catalyst surface can diffuse, mediating the SLG formation at the surface (*J*_G_ = *J*_I_ – *J*_D_). The unsaturated catalyst thus provides a broader processing window, under which SLG can be stabilized before the nucleation of the second layer.

To demonstrate these two scenarios, we grew graphene on polycrystalline films and foils of Co, Cu and Ni following the first-order framework we have developed. We first focus on graphene growth on foils. Fig. 2b–d show the uniform SLG films grown on Ni[25 μm] (Fig. 2b), Co[25 μm] (Fig. 2c) and Cu[25 μm] (Fig. 2d) foils, under conditions optimized for each catalyst. Optical micrographs following transfer to SiO_2_(300 nm)/Si show contrast indicative of uniform monolayer graphene coverage,^33,34^ and the uniformity confirms SLG formation across large areas on each of the catalysts. This is further confirmed by Raman spectra of the graphene grown on the Ni, Co and Cu foils (Fig. 2f) which show the characteristic features of SLG (2D fwhm <40 cm^–1^ and *I*_2D_/*I*_G_ ratio >2), with 2D peaks well fitted with single Lorentzians all with fwhm of ∼35 cm^–1^, and with *I*_2D_/*I*_G_ ratios of 2.8, 3.7 and 3.0 respectively. The *I*_D_/*I*_G_ ratios, which relate to defects within the graphene lattice, are all low with values of Cu (7%), Co (8%) and Ni (10%) and thus indicative of high graphitic quality. We note that the growth conditions under which this uniform SLG is achieved vary greatly for the different catalysts in terms of the temperature, precursor pressure, and exposure time which will be discussed in more detail below.

We now consider the effect of catalyst thickness by investigating thinner catalyst films of Cu[1 μm] (Fig. 2e), Co[250 nm] (Fig. 2g), and Ni[550 nm] (Fig. 2h) exposed to identical conditions as those used for the optimized SLG growth on the respective catalyst foils. After transfer to SiO_2_(300 nm)/Si, optical micrographs of the graphene grown on Co (Fig. 2g) and Ni (Fig. 2h) films show the growth of inhomogeneous few-layer graphene (FLG), with the darker purple regions related to thicker FLG and the white regions to even thicker multilayer graphene. Furthermore, Raman spectra measured across different regions of the samples show variations indicative of the spatial inhomogeneity in the number of graphene layers and their stacking. The typical *I*_2D_/*I*_G_ ratios of <1.5 and upshifted 2D peaks with fwhm of >50 cm^–1^ indicate the FLG grown on both the Co and Ni films is largely turbostratic,^35^ in agreement with previous indications that turbostratic graphene formation may be favoured when there are high concentrations of dissolved carbon near the catalyst surface,^11^ as expected for these carbon saturated films.

For the Cu[1 μm] films, Fig. 2e shows an optical micrograph where some small holes are seen within the graphene film. We note that corresponding holes in the Cu catalyst film are also observed in SEM micrographs of the as-grown graphene (not shown), indicating dewetting of the Cu occurs prior to the graphene deposition. Nevertheless, a continuous graphene film is observed across most of the catalyst film. A representative Raman spectrum of the transferred film shows the characteristic features of SLG, with a 2D peak well-fitted with a single Lorentzian of 34 cm^–1^ fwhm and *I*_2D_/*I*_G_ ratio of 4.6, whilst the negligible D peak confirms high graphitic quality. Raman spectra taken across the sample show similar features confirming that high-quality, uniform SLG is achieved on the Cu[1 μm] films for the same conditions optimized for SLG growth on Cu[25 μm] foils.

**Fig. 3:**
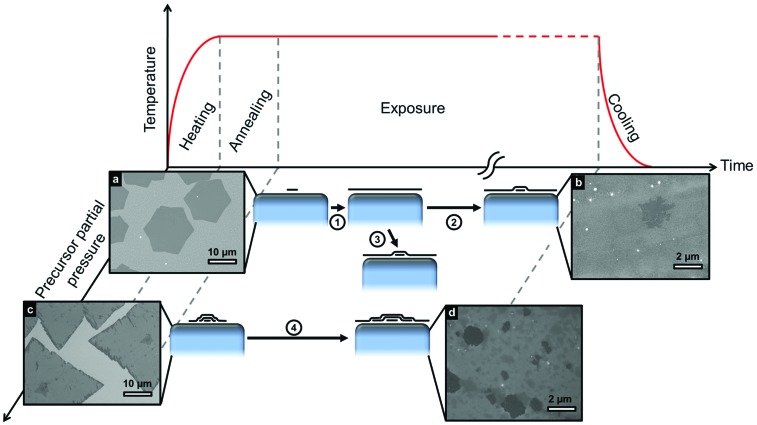
Schematic representation of different routes that lead to the formation of FLG. Temperature profile and growth evolution at different precursor partial pressures are shown. (a–d) Inset SEM micrographs taken after growth on Cu foil 25 μm at the conditions corresponding to their respective schematic. Lower precursor partial pressure promotes the nucleation of SLG islands (a)[1050 °C, ∼50 mbar, 15 min, CH_4_(0.012 sccm)/Ar(250 sccm)/H_2_(26 sccm)], which grow until they coalesce to form a uniform continuous SLG film (step 1). If exposure to hydrocarbon continues (step 2), further layers grow beneath the initial SLG (b) [1050 °C, ∼250 mbar, 200 min, CH_4_(0.012 sccm)/Ar(250 sccm)/H_2_(26 sccm)]. FLG growth can also result from increasing the precursor partial pressure after continuous SLG has formed (step 3). Higher precursor partial pressures at the start of exposure can lead to the direct nucleation of FLG (c) [1050 °C, ∼50 mbar, 2 min, CH_4_(0.15 sccm)/Ar(250 sccm)/H_2_(26 sccm)] with continuing exposure (step 4) resulting in further growth of those layers in contact with the catalyst (d) [1050 °C, ∼50 mbar, 60 min, CH_4_(0.15 sccm)/Ar(250 sccm)/H_2_(26 sccm)].

Despite the demonstration here that SLG can be stabilized across different transition metal catalysts with a broad range of carbon solubilities, we note that FLG can also be produced on these same catalysts under different conditions, even on Cu for which a self-limited growth behaviour is often suggested in the literature.^13,14^ Fig. 3 shows different routes to FLG formation, comparing the evolution of growth at low and high precursor partial pressures with respect to the hydrocarbon exposure time. An initially low precursor partial pressure promotes the nucleation of SLG islands (Fig. 3a) that grow in size with exposure time until they coalesce to form uniform SLG (step 1). Graphene grains of different orientations stitch together forming grain boundaries and defects where they merge.^36^ Prolonged exposure to the precursor (step 2) or an increase in precursor partial pressure (step 3) leads to the nucleation and growth of additional layers beneath the existing SLG^19,37^ resulting in FLG as shown in Fig. 3b. Alternatively, initial exposure to a high precursor partial pressures leads to direct FLG nucleation (Fig. 3c), with the layers in contact with the catalyst continuing to grow in lateral extent with exposure time as shown in Fig. 3d.^38^

Finally, we consider the effect of temperature on the growth outcome, focusing on Co as a representative system (Fig. S1†). Graphene is synthesized on Co using a two-step growth process with an initial hydrocarbon exposure pressure of ∼10^–6^ mbar for 15 min followed by an increase in pressure to ∼10^–5^ mbar for 5 min. On varying the exposure temperatures from 400 °C to 800 °C (following identical pre-treatments at 800 °C), we observe a general improvement in graphitic quality with increasing exposure temperature. At higher temperature however, the improvement in graphene quality is accompanied by the formation of additional graphene layers with inhomogeneous FLG coverage observed (Fig. S1i†). The growth temperature of 700 °C is thus identified as a suitable compromise between achieving high-quality yet still uniform SLG coverage for the Co[25 μm] catalyst foils. On Ni catalysts, high-quality graphene growth can be achieved at slightly lower temperatures (600 °C) which may be desirable for direct integration.^19^

## Discussion

On the basis of our results and existing literature, we now rationalize the observed growth behaviour and develop a first-order model for graphene CVD on catalyst surfaces that can be applied generally to transition metal catalysts. We focus on isothermal growth, as despite the non-negligible carbon solubility of Ni and Co, our extensive *in situ* studies of graphene growth^11,23,24,39–41^ alongside other reports in literature^42–46^ have shown isothermal growth to be dominant for the catalysts and conditions used herein, with the contributions form precipitation on cooling typically being only minor as a result of the rapid decrease in carbon diffusivity with temperature.^24^ During isothermal growth, the supply or removal of carbon at the catalyst surface occurs *via* the gas phase by precursor dissociation or reactive etching by constituents of the growth atmosphere^24,47,48^ (*e.g.* oxygen, hydrogen, water), as well as by diffusion into or out of the catalyst bulk.^19,41^ Typical graphene CVD processes adopt a pre-treatment phase to reduce carbon contamination within the catalyst and thus provide a more defined starting point prior to growth. The gas-phase supply/removal of carbon is complex, affected by parameters such as temperature, catalyst activity, precursor/etchant chemistry, pressure and the boundary layer that can be present for growth conditions with higher total pressures.^49^ Nevertheless, the growth atmosphere is typically adjusted to deliver a net flux of carbon to the catalyst surface, with graphene growth at the catalyst surface (*J*_G_) fed by the balance between this gas-phase supply (*J*_I_) and diffusion into the catalyst bulk (*J*_D_). Although different hydrocarbon precursors are selected here for each catalyst (C_2_H_2_ for Co and Ni, CH_4_ for Cu), this is to ensure sufficient catalytic dissociation at the chosen growth temperature whilst avoiding undesired pyrolytic dissociation, and is not observed to significantly alter the growth evolution otherwise. For graphene grown on Cu, CH_4_ is mixed with Ar and H_2_ during the exposure period. An inert buffer gas (*e.g.* Ar) is used in conjunction with CH_4_ because it allows the dilution of carbon required without the need for stringent pumping (*i.e.* a large Ar background means gas partial pressure rises slowly). H_2_ is expected to participate more actively in reactions occurring at the catalyst–gas interface and it has been suggested to play a key role in limiting the formation of copper oxide at the catalyst surface,^50^ helping to activate surface bound carbon needed for growth^51^ and promoting the desorption/etching of small active carbon species.^51^ The detailed role of the CH_4_/H_2_ balance in the growth reactions has been discussed in previous reports, on the basis of adsorption modelling^52^ and thermodynamic analysis.^53^

At the start of precursor exposure there is an incubation period during which *J*_I_ = *J*_D_, and the local concentration of carbon at the surface increases until it reaches the catalyst's solubility limit and a local supersaturation (Δ*c*) develops. Graphene islands subsequently nucleate and grow with continuing exposure (*J*_G_ = *J*_I_ – *J*_D_) until they eventually impinge on one another to form a complete graphene layer with grain boundaries and defects where the graphene grains merge.^36^ Despite the graphene coverage, carbon continues to be supplied to the catalyst surface through leakage pathways such as defects and grain boundaries, causing the carbon concentration at the catalyst surface to again increase until additional graphene layers nucleate (Fig. 3) beneath the existing layer.^19^

This general growth evolution has been observed for Ni^19,23,24^ and Cu^39,50^ in our previous work and is now also confirmed for Co. Nevertheless, significant variations in growth outcome are reported in literature for these different catalysts under various process conditions, and indeed our own results here show notable effects of catalyst thickness, precursor pressure, and exposure time (see Fig. 1 and 2). This highlights the importance of kinetic factors in determining the growth outcome, particularly in relation to carbon delivery to and removal from the catalyst surface, and the nucleation of new graphene islands/layers or incorporation into existing graphene islands. Considering a simple kinetic model for the evolution of graphene coverage for a single crystal sample of infinite thickness based on the balance of carbon fluxes at the catalyst surface (Fig. 1b) reveals a broad plateau in the exposure times over which close to SLG coverage is achieved (red line in Fig. 2a).^19^ This results from the reduction in carbon supply to the surface with increasing graphene coverage combined with the continuing diffusion of carbon into the catalyst bulk, meaning that closure of the film is gradually approached, whilst extended exposure is required to develop the supersaturation necessary for additional layer formation (Fig. 3b). The stabilization of SLG is thus achieved by locally filling the catalyst with carbon close to its surface, whilst avoiding saturating the whole catalyst with carbon throughout its thickness, so that the catalyst bulk continues to provide a mediating sink for carbon to diffuse into. We note that some supply of carbon to the catalyst through the existing graphene layers is key to the observed merging of domains to form a continuous film, as otherwise SLG coverage would only be asymptotically approached.

This model remains insightful when considering growth on more economically realistic polycrystalline catalysts such as those studied here. The broad plateau is key to stabilizing SLG across different catalyst grains, on which the graphene coverage evolves at different rates due to orientation dependent variations in precursor dissociation and graphene nucleation barrier. Indeed, our results in Fig. 2 demonstrate that uniform SLG coverage can be achieved on Co, Cu and Ni polycrystalline catalysts, by controlling the CVD conditions and catalyst thickness to avoid saturating the catalyst with carbon throughout its thickness during growth.

For a finite catalyst film that becomes saturated with carbon throughout its thickness during growth, the width of the monolayer plateau shrinks significantly, as the catalyst bulk no longer provides a mediating carbon sink (Fig. 2). Instead, the formation of inhomogeneous FLG readily occurs (Fig. 3), as we observe for thinner catalyst films of Co and Ni for the same exposure conditions under which uniform SLG is formed on much thicker foils (Fig. 2g and h). This further highlights that isothermal growth is the dominant growth process, as the optical images (Fig. 2g and h) indicate that the average FLG thicknesses (>2 layers for Co[250 nm] and >5 layers for Ni[550 nm]) are significantly higher than expected by precipitation alone, based on the solubility limits and thicknesses of the catalyst films: ∼0.3 layers for Co[250 nm]^25,54^ and ∼2.8 layers for Ni[550 nm]^26^. Interestingly, for the thin Cu film, uniform SLG is still formed under the same conditions as for the foil, indicating that in spite of its reduced thickness the catalyst does not become saturated with carbon throughout. This shows a broad processing window exists over which SLG can be stabilized on Cu catalysts; nevertheless, the growth of FLG on Cu is possible as shown in Fig. 3. Exposure to high initial partial pressure leads to a quick inhomogeneous supersaturation of the catalyst surface with carbon, nucleating FLG from the beginning of the process (Fig. 3c). Alternatively, the prolonged exposure of Cu to hydrocarbon or the increase in precursor partial pressure after SLG growth leads to the formation of additional graphene layers (Fig. 3b) as steps 1,2 or 1,3 respectively show in the schematics of Fig. 3. This results from the continuing supply of carbon to the catalyst through defects and/or grain boundaries, which increases the carbon concentration at the catalyst surface until additional graphene layers nucleate under the existing SLG film (Fig. 3).

Whether or not a catalyst becomes saturated with carbon throughout its thickness prior to complete SLG coverage depends on the rate at which carbon is delivered to the catalyst (*J*_I_), the catalyst thickness (*l*) and its permeability (*P*). The relation between these parameters is explained in detail by Weatherup *et al.* ^19^ and it provides a lower bound for the thickness required to avoid catalyst saturation prior to complete SLG coverage given by:1
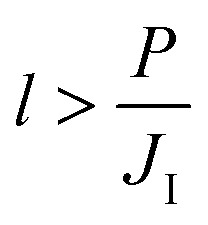



This inequality indicates that a low value of *J*_I_, relative to *l*, should be avoided, as otherwise the catalyst is slowly filled with carbon throughout its thickness becoming saturated with carbon prior to graphene nucleation and thus yielding inhomogeneous FLG. Higher exposure pressures corresponding to higher values of *J*_I_ are instead preferred, but we note that too high a carbon flux leads to rapid filling of the catalyst surface and a large carbon supersaturation developing prior to nucleation. This favours a high nucleation density and thus small grain sizes, as well as the direct nucleation of FLG islands (Fig. 3c). For this reason the growth pressure at which SLG is achieved on Ni herein is close to the lowest required to form complete SLG at the selected growth temperature, without saturating the catalyst throughout.^19^ This also motivates the two-step process^55^ adopted here for the growth on Co, where a low initial exposure pressure achieves a reduced nucleation density whilst the subsequent higher pressure exposure ensures a closed film before the catalyst becomes saturated with carbon throughout its thickness. Indeed we find that for both Ni and Co, the longer growth times needed for complete coverage at lower growth pressures result in inhomogeneous FLG formation on numerous regions of the catalyst. The inequality of (1) can also account for the different behaviour of Cu in comparison to the Ni and Co, when the catalyst thickness is reduced (Fig. 2). Whilst Ni at 600 °C has a higher carbon solubility than Co at 700 °C, their carbon permeabilities are of similar magnitude (Ni at 600 °C ∼4 × 10^12^ atoms per m per s and Co at 700 °C ∼2 × 10^13^ atoms per m per s),^25,26,54^ based on the values of diffusivity available in literature,^25,26,54,56,57^ and indeed both catalysts show a similar transition from uniform SLG formation to inhomogeneous FLG formation as the catalyst thickness is reduced. There is little data available on the diffusivity of carbon in Cu,^27^ and reported values of carbon solubility at 1000 °C vary between 0.0007 atom% and 0.0280 atom%,^27,28^ however the lack of the transition from uniform SLG formation to inhomogeneous FLG formation indicates Cu has a significantly lower carbon permeability under these conditions. We therefore suggest that the broad processing window for SLG formation on Cu is related to this low permeability (relative to the rate of carbon delivery to the catalyst surface), and is not solely the result of the low carbon solubility of Cu. We note that catalyst alloying provides new opportunities for rational catalyst design by allowing properties such as permeability to be tuned to obtain a desired outcome by matching the catalyst with the growth process.

The improvement in graphene quality observed with increasing growth temperature (Fig. S1†) can also be understood by consideration of kinetic factors. Whether the carbon delivered to the catalyst surface is incorporated into an existing island or a sufficient local supersaturation develops to nucleate a new graphene island, depends on how readily carbon is transported across the catalyst surface. This transport is expected to be dominated by surface diffusion, given this is typically much faster than other possible routes such as grain boundary or bulk diffusion.^58–60^ The increase in graphene domain sizes at higher growth temperatures, and improved graphitic quality, is thus attributed to the increase in surface diffusivity with temperature.^41^ We further note that increased catalytic dissociation and increased probability of defect healing may also contribute to this improvement in quality observed for higher growth temperatures.^61^ This might suggest that higher growth temperatures are in general preferable, however this must be balanced against the increased likelihood of saturating the catalyst throughout with carbon due to the increase in permeability with temperature, as revealed by the inhomogeneous FLG growth observed at 800 °C on Co (Fig. S1i†). The potential for catalyst sublimation, and precursor pyrolysis at higher temperatures must also be considered as well as restrictions on temperature associated with the direct integration of graphene into device structures. We note that we have previously reported a similar variation in quality with temperature both Cu^50^ and Ni^23,41^ where in the case of Ni, graphene can be synthesised at even lower temperatures (600 °C) without compromising quality.^19^ Further reductions in growth temperature to those compatible with back-end CMOS integration (≤450 °C) can be achieved with Au–Ni alloy catalysts, whilst still maintaining reasonable graphene quality.^23,62^

The catalysts considered herein (Co, Ni and Cu), all present simple bulk phase diagrams for the growth temperatures used, consisting of only graphite and a metal-carbon solid-solution, without the involvement of other bulk intermediate phases^10,23,31^ as schematically indicated in Fig. 1a. However, other transition metal catalysts present more complex phase diagrams, such as Fe where the coexistence of different phases further complicates the growth model. Nevertheless the first-order model developed here can serve as a starting point for understanding these more complex systems, even though adjustments for the presence of other phases may be necessary, and indeed we have recently found through this approach that under suitable conditions SLG can be stabilized on Fe.^63^

## Conclusions

In summary, we have developed a first-order model for graphene growth on transition metal catalysts with which we rationalise our systematic CVD calibrations for high-quality uniform SLG on Co, Ni, and Cu. We thereby identify key CVD process parameters (temperature, precursor pressure, exposure time) that must be adjusted to achieve the desired outcome based on consideration of the catalyst properties (permeability, thickness) and the kinetics of growth. SLG and FLG formation on all of these catalysts occurs predominantly at temperature during precursor exposure (isothermal growth) for the process conditions adopted herein, rather than by precipitation on cooling. The simple distinction previously made in literature between catalysts with low carbon solubility, where surface segregation/adsorption of SLG is proposed to dominate, and higher carbon solubility catalysts, where FLG formation by precipitation on cooling is assumed, is therefore not supported either by our *in situ* observations of isothermal growth, nor by the extent of FLG formation we observe. Instead, our results indicate a distinction in growth behaviour based on whether a catalyst becomes saturated with carbon throughout its thickness during the growth process. For the conditions at which we obtain high-quality and uniform SLG on Co, Ni, and Cu, we remain in a regime in which the catalyst is not filled with carbon throughout its thickness. The catalyst bulk thus provides a sink into which carbon arriving at the catalyst surface can diffuse, mediating the SLG formation at the surface. For conditions at which the Cu, Ni and Co catalysts become saturated with carbon throughout, the undesired formation of inhomogeneous FLG is instead observed, although the low permeability of Cu leads to a particularly broad window of processing conditions over which SLG can be stabilized.

The understanding developed herein provides important insights into the CVD of graphene on transition metal catalysts. Given the general nature of these insights, we expect them to be relevant to a range of different catalyst materials including alloys,^23,63–66^ and to provide a framework for the rational design of catalysts and processes for achieving graphene with properties tailored to specific applications. We expect this to be important in the design of growth strategies to obtain a desired growth outcome on a specific catalyst material, and/or where constraints are placed on the process conditions that can be used, *e.g.* direct integration into device structures.^9,10^

## Methods

Graphene is synthesized by chemical vapour deposition in a custom-built cold-wall reactor for Co and Ni catalysts whilst a commercially available Aixtron BM Pro (4 inch) machine is used for graphene growth on Cu. Polycrystalline sputter-deposited films (Co[250 nm, 99.995% purity sputter target], Ni[550 nm, 99.995% purity sputter target], Cu[1 μm, 99.99% purity sputter target] as measured by mechanical profilometry) on SiO_2_ and commercially available polycrystalline 25 μm Ni(99.99% purity), Cu(99.999% purity) and Co(99.95% purity) foils are studied.

For graphene grown on Co and Ni, samples are annealed for 15 min in H_2_ (1 mbar) heating at ∼300 °C min^–1^ to the growth temperature. The chamber is then quickly pumped down and once the base pressure ∼10^–6^ mbar is reached, samples are exposed to a hydrocarbon precursor [C_2_H_2_ with pressures in the range 10^–6^–10^–3^ mbar for 5 s to 180 min] and subsequently cooled down to room temperature in vacuum at ∼100 °C min^–1^.

For graphene grown on Cu, samples are annealed for 30 min at 250 mbar in a mixture of H_2_/Ar (50 sccm/200 sccm) heating at ∼100 °C min^–1^ to the growth temperature. CH_4_ diluted 0.1% in Ar is then introduced to the chamber for 180 min promoting growth under a CH_4_/H_2_/Ar (12 sccm/26 sccm/250 sccm) atmosphere and finally cooled down to room temperature in Ar.

As grown graphene is characterised *ex situ* using scanning electron microscopy (SEM, Carl Zeiss SIGMA VP, 1.5 kV). When imaging graphene with secondary electrons (SE), it generally appears darker than the catalyst surface due to the low generation of SE in graphene.^67^ The greater the number of graphene layers the darker they appear. Electron channelling contrast (arising from different grain orientations in the polycrystalline catalyst) can also be seen from the variations in contrast within the graphene regions in SEM images. Optical microscopy and Raman spectroscopy (Renishaw Raman InVia microscope, 457 nm wavelength with 1 mW on the sample, 50× objective) are performed after graphene is transferred to SiO_2_(300 nm)/Si substrates and corroborate the SEM results.

Graphene transfer is performed by depositing polymer supports [polymethylmethacrylate (PMMA) or polystyrene (PS)] onto the graphene/metal-catalyst sample. Wet etching techniques are employed to remove the metal. For graphene grown on Cu, 0.5 M FeCl_3_ or 0.5 M (NH_4_)_2_S_2_O_8_ aqueous solutions are used as etchants and for graphene grown on Co 10 M HCl acid is used. Graphene synthesized on Ni catalysts is transferred using an electrolysis-based bubbling technique in an aqueous NaOH (1 M) solution.^20,68^ The graphene/polymer film is rinsed in DI water and then transferred to SiO_2_(300 nm)/Si substrate. The polymer support is subsequently removed by immersion in a suitable solvent [acetone for PMMA and ethyl acetate for PS] followed by a bath in IPA and drying with an N_2_ flow.
