# Supplementary material for: Towards a general growth model for graphene CVD on transition metal catalysts
Source: Nanoscale. 2016 Jan 5;8(4):2149–58. doi: 10.1039/c5nr06873h (PMC4755235; doi:10.1039/c5nr06873h)
Supplement: Supplementary file 1 [file NR-008-C5NR06873H-s001.pdf]

Electronic Supplementary Material (ESI) for Nanoscale.
This journal is © The Royal Society of Chemistry 2015

Electronic Supplementary Information:

Towards a General Growth Model for Graphene CVD on Transition Metal Catalysts

Andrea Cabrero-Vilatela, [a] *Robert S. Weatherup,**[a] *Philipp Braeuninger-Weimer,* [a] *Sabina Caneva*[a] *and Stephan Hofmann*[a]
[a]Department of Engineering, University of Cambridge, Cambridge CB3 0FA, United Kingdom
* Email: rsw31@cam.ac.uk

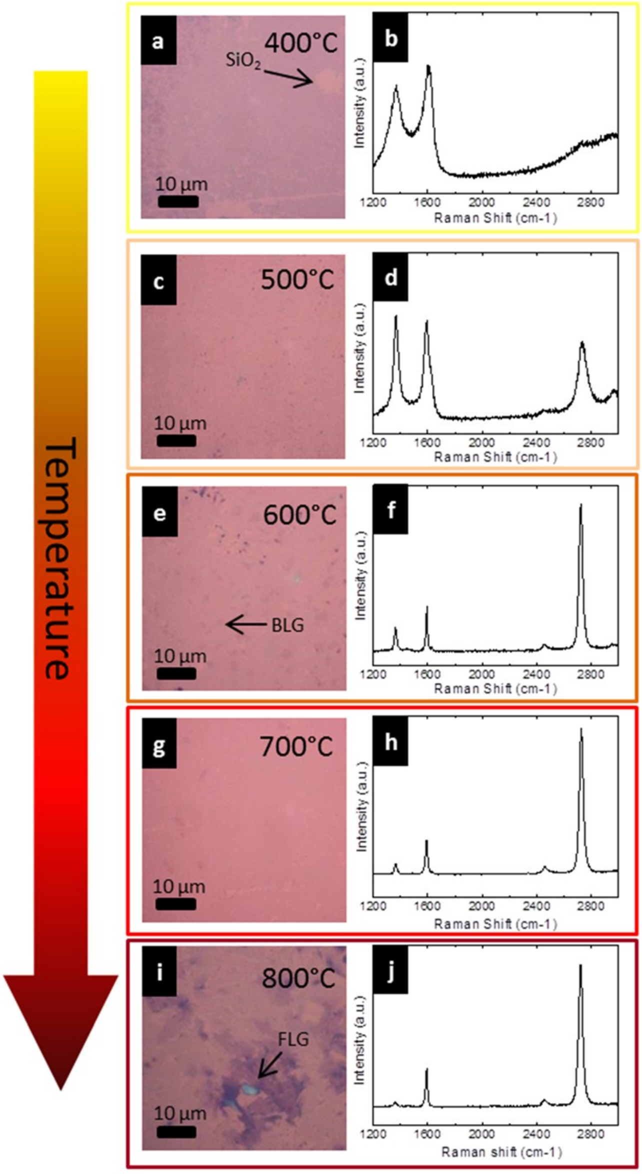

Figure S1: Raman spectra and optical images of graphene grown on 25µm Co foil with C_2H_2 two stage growth exposures (10^{-6} mbar 15 min then 10^{-5} mbar 5min) at different temperatures.
